# Development of an Immunochromatographic Test with Recombinant MIC2-MIC3 Fusion Protein for Serological Detection of *Toxoplasma gondii*

**DOI:** 10.3390/vetsci12060509

**Published:** 2025-05-22

**Authors:** Jianzhong Wang, Yi Zhao, Jicheng Qiu, Jing Liu, Rui Zhou, Xialin Ma, Xiaojie Wu, Xiaoguang Li, Wei Mao, Yiduo Liu, Heng Zhang

**Affiliations:** 1 Shanxi Key Laboratory for Modernization of TCVM, College of Veterinary Medicine, Shanxi Agricultural University, Jinzhong 030810, China; jianzhongwang@cau.edu.cn (J.W.); vm202420402@sxau.edu.cn (J.L.); vm202430960@sxau.edu.cn (R.Z.); 2 Laboratory of Veterinary Clinical Pharmacology, College of Veterinary Medicine, Inner Mongolia Agricultural University, No. 29, Saihan District, Hohhot 010011, China; zoey18047287583@163.com (Y.Z.); maowei2014@imau.edu.cn (W.M.); 3 Beijing Yuanda Xinghuo Medicine Technology Co., Ltd., Beijing 102615, China; qiujicheng2011@outlook.com; 4 Hangzhou Evegen Biotech Co., Ltd., Hangzhou 310018, China; 18862236726@163.com (X.M.); wusxy2006@163.com (X.W.); lxg@aijinbio.com (X.L.); 5 Department of Animal Science and Technology, Beijing Vocational College of Agriculture, Beijing 102442, China; 6 Jiangsu Agri-animal Husbandry Vocational College, Taizhou 225300, China

**Keywords:** *Toxoplasma gondii*, immunochromatographic test, antibodies against *T. gondii*, MIC2-MIC3 fusion protein, serological detection

## Abstract

*Toxoplasma gondii* is a globally significant zoonotic pathogen responsible for severe parasitic diseases in humans and animals. This study aimed to design, develop, and evaluate a novel immunochromatographic test (ICT) using a recombinant MIC2-MIC3 fusion protein (rMIC2-MIC3) for detecting specific antibodies against *T. gondii*. The ICT demonstrated exceptional sensitivity, capable of detecting *T. gondii*-specific antibodies in sera diluted up to 1:8. Specificity evaluation confirmed no cross-reactivity with antibodies against other parasites, such as *Neospora caninum, Cryptosporidium suis, Eimeria tenella*, and *Sarcocystis tenella*. Stability tests revealed the test strips maintained full functionality after 12 weeks of storage at 24 °C. The coincidence rate of the colloidal gold test strips prepared in this study with a commercial ELISA kit was 94.59%. Comparisons with advanced serodiagnostic tools, such as chimeric antigen-based ELISAs and recombinant protein diagnostics, further highlighted its robustness and applicability. These findings underscore the potential of the rMIC2-MIC3-based ICT as a reliable, economical, and accessible diagnostic tool for toxoplasmosis in veterinary and human medicine.

## Simple Summary

*Toxoplasma gondii* is a widespread parasite that affects animals and humans, causing health and economic challenges. This study developed a rapid diagnostic tool, an immunochromatographic test (ICT), using a combined MIC2-MIC3 protein to detect *T. gondii* antibodies in cats. The test was optimized and evaluated using clinical feline serum samples and control sera from other parasitic infections, such as *Neospora caninum* and *Sarcocystis tenella*, to ensure accuracy. The ICT showed high sensitivity (100%) and specificity (85.7%) in clinical evaluations compared to a commercial ELISA kit, with no cross-reactivity observed in specificity tests with other parasites. Its quick 15 min results and portability make it suitable for use in farms, clinics, or disease outbreak investigations.

## 1. Introduction

*Toxoplasma gondii* (*T. gondii*) is an obligate intracellular protozoan parasite widely distributed across the globe, causing zoonotic parasitic diseases [1]. *Toxoplasma gondii* is capable of infecting both humans and animals via waterborne transmission, direct contact, or transplacental transmission. It is estimated that approximately one-third of the global population is infected with this parasite [2].While most infections are asymptomatic, some infections in immunocompromised individuals or cases of congenital infection can lead to life-threatening complications such as hydrocephalus, retinochoroiditis, and miscarriage [3,4]. The parasite also significantly impacts the livestock industry, leading to abortions, stillbirths, and neonatal deaths in animals, particularly sheep and goats [5]. Considering the public health risks and economic consequences posed by this zoonotic disease, it is necessary to develop sensitive and specific diagnostic tests for managing toxoplasmosis.

The parasite *T. gondii* can invade both non-phagocytic cells and phagocytic cells of mammals. Non-phagocytic cells do not possess the ability to actively ingest or phagocytize. Therefore, *T. gondii* exploits its distinctive gliding motility for the invasion of non-phagocytic cells [6]. Gliding and invasion are contingent upon an actin-myosin system, as well as the processes of protein release from apical secretory vesicles termed micronemes and the subsequent capping of these proteins. Microneme proteins (MICs) encompass modules that are homologous to the adhesive domains of proteins from higher eukaryotes, which have been demonstrated to bind to receptors on host cells. Apart from their binding to host cells, a key function of MICs is to serve as a bridge for the extracellular attachment between host cells and the intracellular actin-myosin motility apparatus [6].

It has been demonstrated that the MICs play a primary and significant role in virulence and pathogenicity, including MIC1-MIC12, AMA1, M2AP, PLP1, ROM1, SPATR, SUB1, and TLN4 [6,7,8]. The principal adhesin orthologs in *T. gondii* are identified as microneme protein 2 (MIC2). MIC2 is part of a family of parasite adhesins called TRAP proteins. These TRAP proteins possess a widely recognized integrin I/A-domain, which is present across diverse taxa, including prokaryotes, protozoa, plants, and metazoan animals. MIC2 plays a pivotal role in *T. gondii* cell invasion/penetration by forming a complex with MIC2-associated protein (M2AP) to mediate adhesion to host cells and facilitate gliding motility, a prerequisite for successful invasion [6]. MIC2 has the ability to interact with extracellular ligands through its ectodomains and simultaneously link to the motility apparatus via its cytoplasmic domains. This dual functionality endows *T. gondii* with the capabilities of gliding motility and host cell invasion.

Microneme protein 3 (MIC3) is among the most commonly employed proteins in the realm of vaccine development for *T. gondii* [7]. As a crucial vaccine candidate, MIC3 is capable of eliciting a robust and durable humoral as well as cellular immune response. MIC3 demonstrates a high-affinity binding to host cells, and its receptor-binding site is intricately associated with the N-terminal chitin-binding-like (CBL) domain. This domain likely contributes significantly to the protein’s function in host–pathogen interactions, potentially through mediating the initial attachment of the parasite to the host cell surface. Furthermore, MIC3 has been detected to be expressed across three parasitic life stages of *T. gondii*, namely tachyzoites, bradyzoites, and sporozoites [9]. Notably, while microneme proteins are generally not abundantly expressed during the sporozoite stage, MIC3 defies this general trend.

Human infection with *T. gondii* typically occurs through two main routes [3]. One is the ingestion of oocysts that are excreted in cat feces. The other is the consumption of meat from infected animals, which may contain long-lived tissue cysts. Consequently, immunization with MIC3 elicits a specific immune response that may prevent the release of tachyzoites from cysts (housing bradyzoites) and mature oocysts (enclosing sporozoites). Bioinformatics analysis shows that MIC3 has favorable immunogenicity and antigenicity indices, indicating its potential as a vaccine candidate against related parasitic infections [6,7,8].

Conventional immunological diagnostics, such as enzyme-linked immunosorbent assays (ELISA), indirect fluorescent antibody tests (IFAT), latex agglutination tests (LAT), and indirect hemagglutination tests (IHA), have proven effective but come with limitations. These include the need for expensive equipment, well-trained personnel, and laboratory infrastructure, making them less accessible in resource-constrained areas [10,11]. In response, immunochromatographic tests (ICTs) have emerged as a well-established point-of-care diagnostic technique. The most widely used format is colorimetric detection employing gold nanoparticles, offering rapid and cost-effective solutions for field applications [12,13].

Despite these advancements, challenges persist with ICTs based on surface antigens [14,15] or dense granule proteins [16,17]. Variability in antigen expression across different infection stages and parasite strains limits the diagnostic accuracy of these methods [18,19]. Therefore, identifying and evaluating additional diagnostic antigens is essential to optimizing *T. gondii* detection.

MICs have emerged as optimal candidates for ICT development. MIC3, in particular, exhibits strong immunogenicity and is expressed consistently across tachyzoite, bradyzoite, and sporozoite stages [9]. Additionally, antigenic regions of MIC2 have demonstrated protective immunity in animal models [20]. Leveraging the complementary properties of MIC2 and MIC3, this study developed a recombinant MIC2-MIC3 fusion protein and evaluated its performance in a one-step ICT for the serological detection of *T. gondii* in various animal serum samples.

## 2. Materials and Methods

### 2.1. Preparation of the Recombinant Proteins

The MIC2 gene (TGGT1_201780) and MIC3 gene (TGGT1_319560) of *T. gondii* were designed based on the protein sequence from the NCBI Gene Bank. After analyzing the hydrophilicity and hydrophobicity of the protein using ExPASy-ProtScale, the MIC2 (267–345 aa) sequence and the MIC3 (234–307 aa) sequence were selected from the region predicted to have a relatively high hydrophilic content for fusion. The two proteins were linked by the flexible peptide GSGSG. The amino acid sequence of the fusion protein was converted into a nucleotide sequence and codon-optimized based on E. coli preferences. The coding sequences of MIC2 (267–345 aa)-GSGSG-MIC3 (234–307 aa) fusion protein were biosynthesized by Sangon Biotech (Shanghai, China) Co., Ltd. These sequences were cloned into the pET-32a vector (Novagen, Darmstadt, Germany) for expression. The recombinant protein was generated by transfecting pET-32a-MIC2-MIC3-His into *E. coli* Transetta (DE3). Proteins fused with His-tags were purified by affinity chromatography using Ni-IDA agarose (QIAGEN, Hilden, Germany) according to the manufacturer’s instructions. Then, the collected elution buffer containing the recombinant protein was dialyzed to remove imidazole and exchanged into phosphate buffer (PBS, pH 7.4).

The purified MIC2-MIC3 recombinant protein was then verified by SDS-PAGE and Western blot with rabbit anti-*T. gondii* serum. Briefly, purified protein samples (10 µg) were separated by 10% sodium dodecyl sulfate-polyacrylamide gel electrophoresis (SDS-PAGE) under reducing conditions using a Mini-PROTEAN Tetra System (Bio-Rad, Hercules, CA, USA). Proteins were transferred onto a polyvinylidene difluoride (PVDF) membrane (0.45 µm, Millipore, Burlington, MA, USA) using a wet transfer system (Bio-Rad) at 100 V for 1 h in transfer buffer (25 mM Tris, 192 mM glycine, 20% methanol, pH 8.3). The membrane was blocked with 5% non-fat dry milk in Tris-buffered saline with 0.1% Tween-20 (TBST) for 1 h at room temperature. Rabbit anti-T. gondii serum (1:100) was diluted in TBST with 5% milk and incubated with the membrane overnight at 4 °C. After three 5 min washes with TBST, the membrane was incubated with horseradish peroxidase (HRP)-conjugated secondary antibodies (goat anti-rabbit IgG, 1:5000, Sigma-Aldrich, St. Louis, MO, USA) for 1 h at room temperature. Following three additional TBST washes, protein bands were visualized using an enhanced chemiluminescence (ECL) detection system (Amersham ECL Prime, GE Healthcare, Chicago, IL, USA) and imaged.

To prepare the strip, the purified MIC2-MIC3 recombinant protein was then fixed on a nitrocellulose filter membrane (NC membrane) as a capture reagent (test line).

### 2.2. Preparation of Gold Conjugated Protein A

All glassware used for the preparation was pretreated with Sigmacote to prevent adsorption and thoroughly cleaned with aqua regia [HNO_3_/HCl (*v*/3*v*)] prior to use. Colloidal gold particles were synthesized following established protocols [21]. Briefly, 2.5 mL of 1% trisodium citrate solution was added to 100 mL of boiling 0.01% HAuCl_4_ solution under continuous stirring. The solution was boiled for an additional 10 min until the color transitioned from blue to dark red, indicating successful colloidal gold formation. After removing the heat source, the solution was stirred for another 5 min to stabilize the particles. The resulting gold colloid, supplemented with 0.01% (*m*/*v*) sodium azide (NaN_3_), was stored in a dark container at 4 °C.

To prepare gold-conjugated Protein A, 1 mL of Protein A solution (1.0 mg/mL) was gently mixed with 100 mL of the colloidal gold solution under continuous stirring. Bovine serum albumin (BSA) was added to a final concentration of 1% (*w*/*v*) to stabilize and block the conjugated particles. Following centrifugation, the conjugate pellet was resuspended in 0.01 M phosphate buffer solution (PBS) containing 1% (*w*/*v*) BSA, 0.3% (*v*/*v*) Tween-20, 0.9% (*w*/*v*) NaCl, and 0.05% (*w*/*v*) sodium azide. The final preparation was stored at 4 °C.

### 2.3. Preparation of the Immunochromatographic Strip

**Figure 1:**
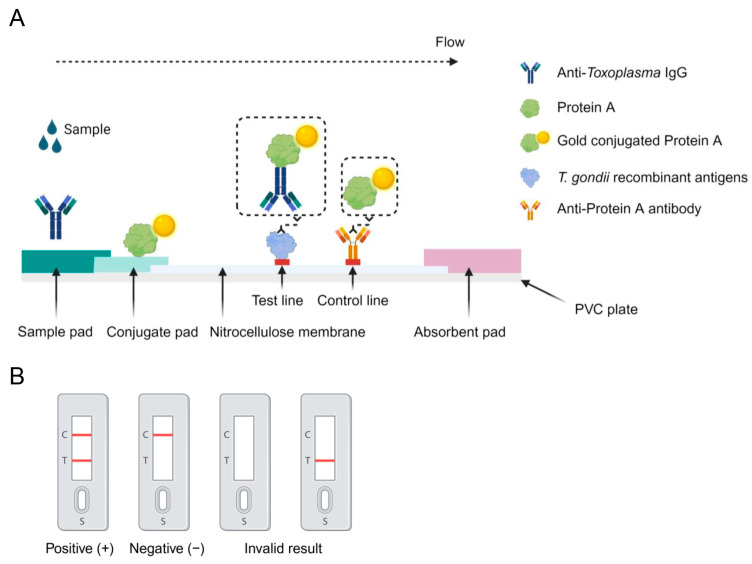
(**A**) Schematic representation of the immunochromatographic strip. Gold-conjugated Protein A reagent was applied to the glass fiber. When a sample containing specific anti-*Toxoplasma gondii* IgG is added to the sample pad, it flows along the strip. Anti-*T. gondii* IgG first forms a complex with the gold-conjugated Protein A, which is then captured by the *T. gondii*-specific recombinant antigens on the test line (T line), resulting in a red band of gold nanoparticles at the T position. Excess gold-conjugated Protein A is subsequently captured by the anti-Protein A antibody on the control line (C line), forming a red band at the C position. (**B**) Illustration of the immunochromatographic test results. A positive result is indicated by the appearance of two red lines at the T line and the C line. A negative result is indicated by the appearance of a single red line at the C line. If only a single red line appears at the T line or if no red lines appear, the test is considered invalid.

The immunochromatographic strip structure is illustrated in Figure 1A. Initially, the sample pad was saturated with a 0.02 M PBS solution (pH 8.5) containing 0.2% Tween-20 and 1.5% (*w*/*v*) BSA. The saturated pad was then dried at 37 °C for 2 h. The colloidal gold probe, consisting of gold-conjugated Protein A, was diluted (1:5, *v*/*v*) with 0.02 M PBS (pH 8.5) containing 5% (*w*/*v*) sucrose and 1.5% (*w*/*v*) BSA. Glass fibers were treated with 0.2% Tween-20 for 12 h and subsequently dried at 37 °C for 1 h before use. The colloidal gold probe was added to the conjugate pad and dried at 37 °C for 1 h.

Diluted recombinant MIC2-MIC3 fusion protein and anti-Protein A IgG were applied onto the nitrocellulose (NC) membrane in a volume of 1 μL/cm to form the test line (T line) and control line (C line), respectively. The NC membrane was treated with 0.02 M PBS containing 1.0% BSA and dried at 37 °C for 2 h. Pure cellulose fiber was used as the absorbent pad.

The test strip components—sample pad, conjugate pad, immobilized NC membrane, and absorbent pad—were assembled onto a PVC plate, as shown in Figure 1A. The assembled strips were cut into 6 mm widths and stored in a desiccator at 4 °C. The possible outcomes of the ICT (positive, negative, and invalid results) are demonstrated in Figure 1B.

### 2.4. Sensitivity, Specificity, and Stability of the Immunochromatographic Test

**Sensitivity Testing:** The positive serum with an ELISA titer of ≥1:1024 was subjected to the sensitivity test. To determine the sensitivity of the ICT, *T. gondii*-positive rabbit serum was serially diluted with 0.01 M PBS at ratios of 1:2, 1:4, and 1:8. Negative rabbit serum served as the control. Both *T. gondii*-positive and negative rabbit sera were preserved in the laboratory. Subsequently, 100 μL of each prepared serum sample was dispensed onto the sample pad. After incubation at room temperature for 10 min, images were captured for analysis. The same procedure was repeated three times with different operators.

**Specificity Testing:** The ICT specificity was evaluated using sera from animals infected with *Neospora caninum*, *Cryptosporidium suis*, *Eimeria tenella, and Sarcocystis tenella*. The results were compared with those obtained from *T. gondii*-positive and negative control sera. There are three positive sera of cats for each pathogen, and the same procedure was repeated three times with different operators. For each test, 100 μL of prepared serum was dispensed onto the sample pad, incubated at room temperature for 10 min, and images were captured for analysis.

**Stability Testing:** ICT strips stored at 24 °C for 12 weeks were tested for sensitivity using diluted *T. gondii*-positive serum of cats to assess their long-term stability. Three replicates of each negative and positive group were compared with the newly prepared test strip for color matching. For each test, 100 μL of prepared serum was dispensed onto the sample pad, incubated at room temperature for 10 min, and images were captured for analysis.

### 2.5. Clinical Evaluation of the Colloidal Gold Test Strip

To confirm the clinical detection effect of the prepared strips, the commercial ELISA kit and colloidal gold test strips were tested in conjunction. Serum samples of cats in this study were from 21 different animal hospitals located in Shanxi, Beijing, Henan, and Zhejiang provinces and were analyzed. A total of 37 clinical feline serum samples were collected from various regions, out of which 23 samples were confirmed to be positive. All samples were stored at 4 °C. The well-received commercial Toxo Test Toxopalsma IgG/IgM Test Kits (Testsealabs, Hangzhou, China) and colloidal gold test strips were tested in conjunction, and the coincidence rate was compared. Serum samples at a dilution of 1:100 with PBST were added to analyze according to the instructions for ELISA kits, while the serum samples were diluted to 1:4 for detection by the ICT strip. All animal-based experimental procedures conducted in the current study were approved by Shanxi Agricultural University and were in accordance with the Guidelines of the Animal Ethical Committee. Signed consent was obtained from all owners.

### 2.6. Statistical Analysis

Each sample was tested in triplicate in this study. The clinical evaluation of the strip and ELISA was compared, and the coincidence rate was calculated as follows: [(true positive + true negative)/(true positive + true negative + false positive + false negative) × 100%].

## 3. Results

### 3.1. Production of MIC2-MIC3 Fusion Protein

**Figure 2:**
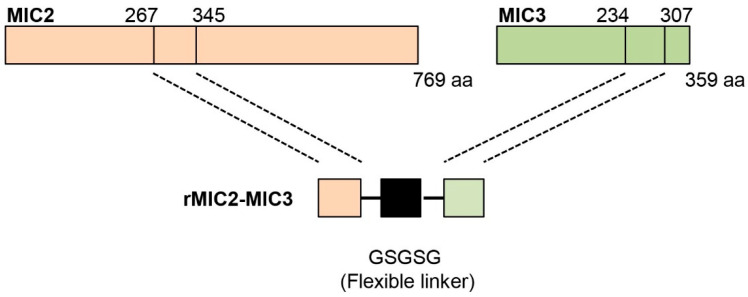
Schematic diagram of the MIC2-MIC3 fusion protein. The MIC2-MIC3 fusion protein was constructed using amino acids 267–345 from MIC2 (769 amino acids) and amino acids 234–307 from MIC3 (359 amino acids), connected with a flexible GSGSG linker polypeptide.

**Figure 3:**
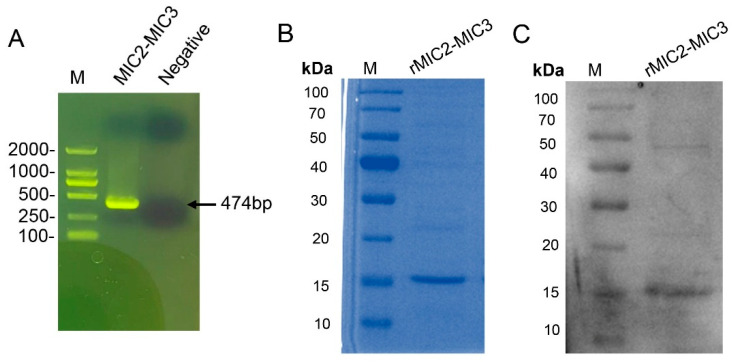
Preparation of the recombinant MIC2-MIC3 fusion protein. (**A**) Agarose gel electrophoresis was used to detect the biosynthetic MIC2-MIC3 DNA fragments. (**B**) Purified MIC2-MIC3 fusion protein was analyzed by SDS-PAGE. (**C**) Purified MIC2-MIC3 fusion protein was analyzed by Western blot (original WB figures see Supplementary Materials).

The MIC2-MIC3 fusion protein was constructed using amino acids 267–345 of MIC2 and 234–307 of MIC3, linked with a flexible GSGSG polypeptide linker (Figure 2). The 474 bp DNA sequence encoding MIC2-MIC3 was biosynthesized (Figure 3A) and cloned into the pET-32a vector for protein expression. DNA sequencing confirmed the successful construction of the recombinant plasmid pET-32a-MIC2-MIC3. The fusion protein was expressed in *E. coli* Transetta (DE3) after transfection with the plasmid. Following induction with IPTG, the MIC2-MIC3 fusion protein was purified and confirmed via SDS-PAGE, revealing a molecular weight of approximately 17 kDa (Figure 3B). Western blotting analysis revealed that the MIC2-MIC3 fusion protein exhibited a clear reaction with anti-*T. gondii* serum (Figure 3C).

### 3.2. Sensitivity, Specificity, and Stability of the Immunochromatographic Test

**Figure 4:**
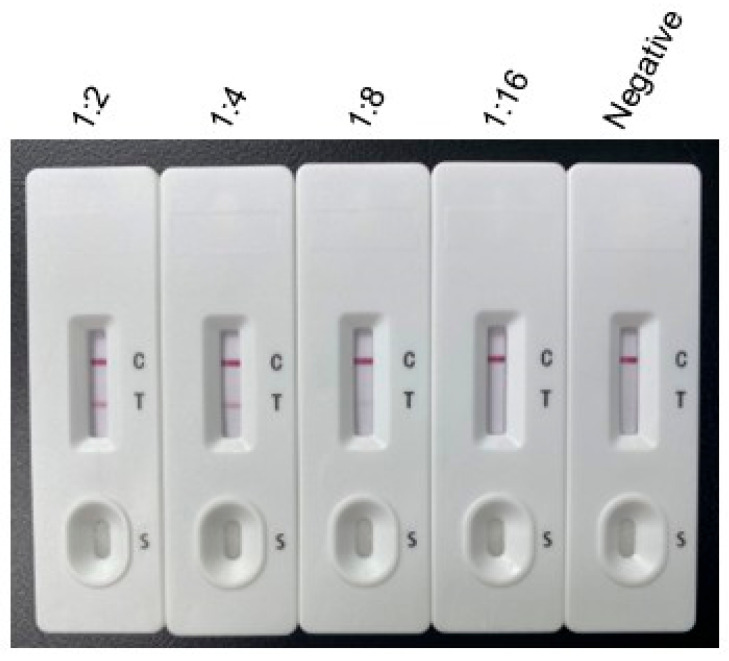
Sensitivity testing of the developed ICT strip. From left to right, the strip was tested using *T. gondii*-positive serum diluted at ratios of 1:2, 1:4, 1:8, and 1:16, followed by standard *T. gondii*-negative serum.

Sensitivity: The sensitivity of the ICT was determined using serial dilutions of *T. gondii*-positive rabbit serum. The results indicated that the test line (T line) was clearly visible when the serum was diluted up to 1:8 (Figure 4), establishing the detection limit at this dilution.

**Figure 5:**
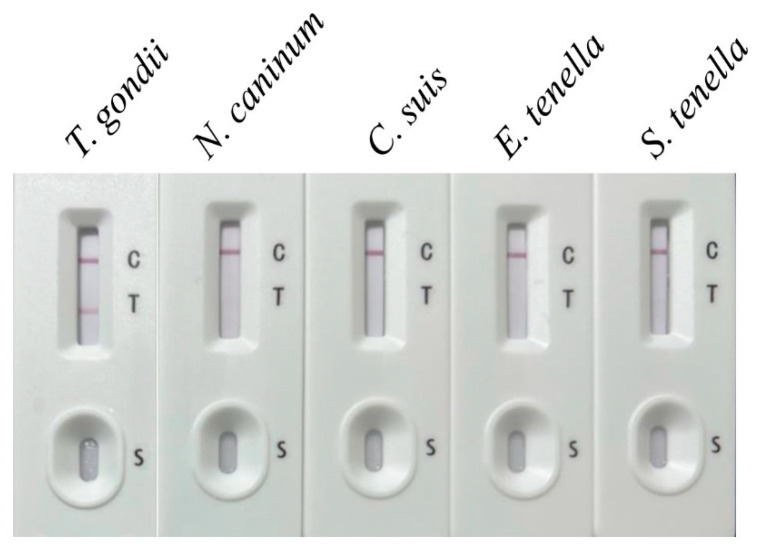
Specificity testing of the developed ICT strip. The strip’s specificity was evaluated using serum samples from *Neospora caninum* (*N. caninum*), *Cryptosporidium suis* (*C. suis*), *Eimeria tenella* (*E. tenella*), and *Sarcocystis tenella* (*S. tenella*). *T. gondii*-positive serum was used as the positive control.

Specificity: Specificity testing was conducted using sera from animals infected with other pathogens, including *Neospora caninum*, *Cryptosporidium suis*, *Eimeria tenella*, and *Sarcocystis tenella*. The ICT showed no cross-reactivity, as only the *T. gondii*-positive serum produced both the test line (T line) and the control line (C line). In contrast, sera from other infections displayed only the control line (C line) (Figure 5).

**Figure 6:**
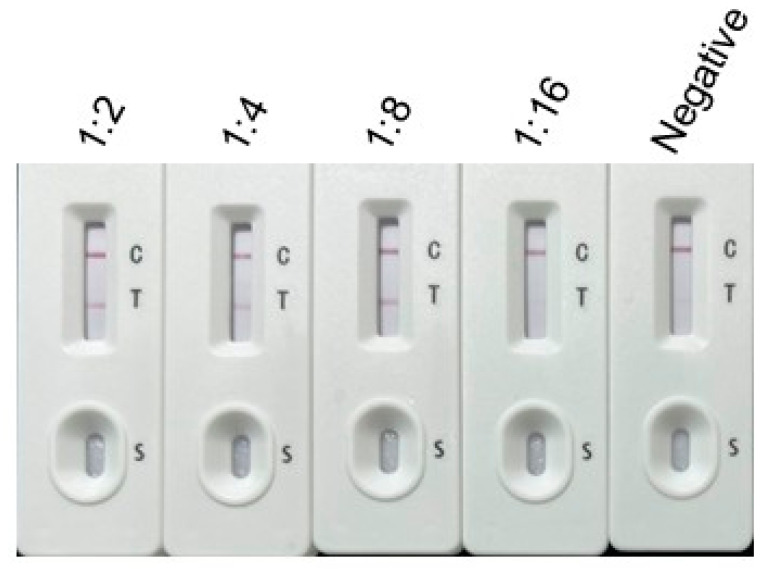
Stability testing of the developed ICT strip. The strips stored at 4 °C for 12 weeks were evaluated. From left to right, the strip was tested using *T. gondii*-positive serum diluted at ratios of 1:2, 1:4, 1:8, and 1:16, followed by standard *T. gondii*-negative serum.

Stability: The stability of the ICT strips was evaluated after storage at 24 °C for 12 weeks. Even after this period, the strips effectively detected *T. gondii*-positive serum diluted to 1:8, with a clear appearance of the T line, confirming the robustness and reliability of the test (Figure 6).

### 3.3. Comparative Analysis with Commercial ELISA Kit

A total of 37 clinical feline serum samples were collected. The commercially available ELISA kit (Abcam, UK) was employed to detect *Toxoplasma gondii* infection in the 37 serum samples, revealing 23 positive and 14 negative samples. Subsequently, these 37 samples were tested using the ICT strips prepared in this study. The results, as shown in Table 1, indicated 25 positive and 12 negative samples. The coincidence rate of the colloidal gold test strips prepared in this study with a commercial ELISA kit was 94.59%, while the sensitivity and specificity of the colloidal gold test strip were 100% and 85.71%, based on the formula in Table 1. This result showed that the ICT strips developed in this study have a good clinical detection effect.

## 4. Discussion

The widespread infectivity and harmfulness of *T. gondii* in humans and animals pose a major epidemiological challenge. Overall, *T. gondii* poses significant risks to both human and animal health, highlighting the need for better control and prevention measures [22]. Over the past few decades, recombinant proteins of *Toxoplasma gondii* have played a pivotal role in enhancing diagnostic tools for serological detection of specific antibodies in human and animal sera [23,24,25,26]. In the current study, we developed a novel ICT employing the MIC2-MIC3 fusion protein as a diagnostic antigen.

Among the MICs, MIC2 and MIC3 exhibit robust immunogenicity and are expressed across all life stages of *T. gondii*, including tachyzoites, bradyzoites, and sporozoites. This study is the first to utilize MIC2-MIC3 fusion protein in an ICT, demonstrating superior diagnostic performance compared to previously studied antigens such as SAG2 and GRA7 [17]. While SAG2 and GRA7 have shown promise, the MIC2-MIC3 fusion protein provides broader antigenic coverage and higher sensitivity [27].

The ICT demonstrated high sensitivity, detecting *T. gondii*-specific antibodies in sera diluted up to 1:8, and showed no cross-reactivity with sera from other parasitic infections (*Neospora caninum*, *Cryptosporidium suis*, *Eimeria tenella*, *Sarcocystis tenella*) in controlled specificity tests [15,28]. In particular, the inclusion of *Eimeria tenella*-positive chicken sera in specificity testing was driven by the close phylogenetic relationship between coccidian parasites like *Eimeria* and *T. gondii*. This allowed us to evaluate the strip’s specificity against antibodies to related parasites that may share homologous MIC2-MIC3 antigens. Furthermore, the ICT was designed with potential applications beyond feline diagnostics, including other animals such as poultry, where Eimeria species are significant pathogens. The absence of cross-reactivity with *E. tenella* sera supports the strip’s specificity for *T. gondii* detection across diverse hosts. In clinical evaluation with 37 feline sera, the ICT achieved a 100% sensitivity and 85.71% specificity compared to a commercial ELISA kit, with a coincidence rate of 94.59% (Table 1). The two false positives observed may result from non-specific binding due to complex sample matrices (e.g., hemoglobin or heterophilic antibodies), though antigenic variation in *T. gondii* strains remains a hypothesis requiring further study. The test strips also maintained functionality after 12 weeks of storage at 24 °C, supporting their practicality for field settings, such as veterinary clinics or farms, where consistent refrigeration may be unavailable [29]. Such stability enables convenient on-site storage and immediate use, facilitating efficient disease detection and control, thus safeguarding animal health and productivity.

To contextualize our ICT’s performance, other methods have been explored. The interferon-gamma release assay (IGRA) is capable of identifying *Toxoplasma gondii* infection as early as four days post-infection (dpi). In contrast, enzyme-linked immunosorbent assay (ELISA) can detect serum IgM and IgG antibodies against *T. gondii* at 10 and 14 dpi, respectively [30]. Nevertheless, it should be emphasized that the ELISA-incorporated IGRA methodology has significant drawbacks, especially in situations where experimental facilities are inadequate, while ICT strips have the advantage of greater simplicity. For sensitivity and specificity, each strip has its own advantages. For instance, an ICT using N-terminal surface antigen 1 (SAG1) linked to granule antigen protein 2 (GRA2) achieved 100% specificity compared to an ELISA with *T. gondii* whole-cell lysates [31]. This suggests strips based on the appropriate antigen could enhance specificity, though differences in reference standards (whole-cell lysate ELISA vs. commercial ELISA kit) limit direct comparisons. The rMIC2-MIC3-based ICT strips demonstrated a detection limit of 1:8 for *T. gondii*-positive serum, which was significantly better than the performance of Rhoptry Protein 14-based strips [28].

The colloidal gold test strip shows promise for *T. gondii* detection in cats, with potential applications in veterinary settings. In high-prevalence regions, its simplicity and 15 min results could enable efficient screening of feline populations, aiding disease control. In low-prevalence areas, it may serve as an initial diagnostic tool for cats with suspected exposure. For vulnerable populations (e.g., pet owners, animal breeders), regular screening could facilitate early intervention. However, these applications require validation with larger, diverse sample sets beyond the 37 feline sera tested here.

However, challenges remain. The accuracy of the test strip may be affected by sample quality and operator proficiency, requiring further validation and optimization. Ensuring consistency with other diagnostic methods, like ELISA and PCR, is essential. Additionally, comprehensive operator training is necessary to standardize usage, result interpretation, and quality control. Despite these challenges, with continuous improvement and quality enhancement, the colloidal gold test strip can play a pivotal role in *Toxoplasma gondii* control in veterinary settings.

Despite these achievements, we acknowledge certain limitations. This study primarily tested 37 clinical feline sera and laboratory-preserved rabbit sera, a relatively small sample size that may not fully represent field diversity. Broader validation with geographically diverse samples, including other host species, is needed for wider applicability. Antibody-based diagnostics, such as ICT, are inherently limited in distinguishing between acute and chronic infections due to the persistence of IgG antibodies [32]. This limitation is particularly pronounced in immunocompromised hosts, where antibody production may be diminished, leading to false negatives [33].

The MIC2-MIC3 fusion protein presents potential for diagnostic applications. Future work will focus on establishing double-antibody sandwich ICTs to improve specificity and differentiate between recent and past infections. Incorporating IgM markers may further enhance early diagnosis capabilities. Additionally, experimental studies will aim to determine the earliest detection time point for *T. gondii* antibodies using ICTs. Testing field samples and exploring multiplexing platforms to detect co-endemic pathogens will also be prioritized to enhance the ICT’s utility [34].

## 5. Conclusions

In summary, the recombinant MIC2-MIC3-based ICT represents a significant advancement in toxoplasmosis diagnostics. Its high sensitivity, specificity, and stability make it a reliable tool for improving disease surveillance and management, particularly in resource-limited settings. Further validation and refinement are necessary to expand its utility and address current limitations. By advancing diagnostic accessibility, this work contributes meaningfully to global efforts in controlling zoonotic diseases.

## Figures and Tables

**Table 1:** Detection of ICT strips.

ICT Strips	Positive	Negative	Total
Test Positive	23 (a)	2 (b)	25
Test Negative	0 (c)	12 (d)	12
Total	23	14	37

Note: The coincidence rate = (a + d)/(a + b + c + d) × 100%. The sensitivity = a/(a + c) × 100%. The specificity = d/(b + d) × 100%.

## Data Availability

The datasets used and/or analyzed during the current study are available from the corresponding author on reasonable request.

## Supplementary Materials

The following supporting information can be downloaded at: https://www.mdpi.com/article/10.3390/vetsci12060509/s1.

## References

[1] Lourido S. (2019). *Toxoplasma gondii*. Trends Parasitol..

[2] Molan A., Nosaka K., Wang W., Hunter M.L.J.T.B. (2019). Global Status of *Toxoplasma gondii* Infection: Systematic Review and Prevalence Snapshots. Trop. Biomed..

[3] Pappas G., Roussos N., Falagas M.E. (2009). Toxoplasmosis Snapshots: Global Status of *Toxoplasma gondii* Seroprevalence and Implications for Pregnancy and Congenital Toxoplasmosis. Int. J. Parasitol..

[4] Montoya J.G., Liesenfeld O. (2004). Toxoplasmosis. Lancet.

[5] Dubey J.P., Jones J.L. (2008). *Toxoplasma gondii* Infection in Humans and Animals in the United States. Int. J. Parasitol..

[6] Liu Q., Li F.-C., Zhou C.-X., Zhu X.-Q. (2017). Research Advances in Interactions Related to *Toxoplasma gondii* Microneme Proteins. Exp. Parasitol..

[7] Rezaei F., Sarvi S., Sharif M., Hejazi S.H., Pagheh A.S., Aghayan S.A., Daryani A. (2019). A Systematic Review of *Toxoplasma gondii* Antigens to Find the Best Vaccine Candidates for Immunization. Microb. Pathog..

[8] Wang J.-L., Zhang N.-Z., Li T.-T., He J.-J., Elsheikha H.M., Zhu X.-Q. (2019). Advances in the Development of Anti-*Toxoplasma gondii* Vaccines: Challenges, Opportunities, and Perspectives. Trends Parasitol..

[9] Wang Y., Yin H. (2015). Research Advances in Microneme Protein 3 of *Toxoplasma gondii*. Parasites Vectors.

[10] Zhang K., Lin G., Han Y., Li J. (2016). Serological Diagnosis of Toxoplasmosis and Standardization. Clin. Chim. Acta.

[11] Robert-Gangneux F., Dardé M.L. (2012). Epidemiology of and Diagnostic Strategies for Toxoplasmosis. Clin. Microbiol. Rev..

[12] Nakayama T., Zhao J., Takeuchi D., Kerdsin A., Chiranairadul P., Areeratana P., Loetthong P., Pienpringam A., Akeda Y., Oishi K. (2014). Colloidal Gold-Based Immunochromatographic Strip Test Comprising Optimised Combinations of Anti-*Streptococcus suis* Capsular Polysaccharide Polyclonal Antibodies for Detection of *Streptococcus suis*. Biosens. Bioelectron..

[13] Meng K., Sun W., Zhao P., Zhang L., Cai D., Cheng Z., Guo H., Liu J., Yang D., Wang S. (2014). Development of Colloidal Gold-Based Immunochromatographic Assay for Rapid Detection of *Mycoplasma suis* in Porcine Plasma. Biosens. Bioelectron..

[14] Huang X., Xuan X., Hirata H., Yokoyama N., Xu L., Suzuki N., Igarashi I. (2004). Rapid Immunochromatographic Test Using Recombinant SAG2 for Detection of Antibodies against *Toxoplasma gondii* in Cats. J. Clin. Microbiol..

[15] Luo J., Sun H., Zhao X., Wang S., Zhuo X., Yang Y., Chen X., Yao C., Du A. (2018). Development of an Immunochromatographic Test Based on Monoclonal Antibodies against Surface Antigen 3 (TgSAG3) for Rapid Detection of *Toxoplasma gondii*. Vet. Parasitol..

[16] Terkawi M.A., Kameyama K., Rasul N.H., Xuan X., Nishikawa Y. (2013). Development of an Immunochromatographic Assay Based on Dense Granule Protein 7 for Serological Detection of *Toxoplasma gondii* Infection. Clin. Vaccine Immunol..

[17] Ybañez R.H.D., Kyan H., Nishikawa Y. (2020). Detection of Antibodies against *Toxoplasma gondii* in Cats Using an Immunochromatographic Test Based on GRA7 Antigen. J. Vet. Med. Sci..

[18] Minot S., Melo M.B., Li F., Lu D., Niedelman W., Levine S.S., Saeij J.P. (2012). Admixture and Recombination among *Toxoplasma gondii* Lineages Explain Global Genome Diversity. Proc. Natl. Acad. Sci. USA.

[19] Ramakrishnan C., Maier S., Walker R.A., Rehrauer H., Joekel D.E., Winiger R.R., Basso W.U., Grigg M.E., Hehl A.B., Deplazes P. (2019). An Experimental Genetically Attenuated Live Vaccine to Prevent Transmission of *Toxoplasma gondii* by Cats. Sci. Rep..

[20] Beghetto E., Nielsen H.V., Del Porto P., Buffolano W., Guglietta S., Felici F., Petersen E., Gargano N. (2005). A Combination of Antigenic Regions of *Toxoplasma gondii* Microneme Proteins Induces Protective Immunity against Oral Infection with Parasite Cysts. J. Infect. Dis..

[21] Liu X., Xiang J.J., Tang Y., Zhang X.L., Fu Q.Q., Zou J.H., Lin Y. (2012). Colloidal Gold Nanoparticle Probe-Based Immunochromatographic Assay for the Rapid Detection of Chromium Ions in Water and Serum Samples. Anal. Chim. Acta.

[22] Dakroub H., Sgroi G., D’Alessio N., Russo D., Serra F., Veneziano V., Rea S., Pucciarelli A., Lucibelli M.G., De Carlo E. (2023). Molecular Survey of *Toxoplasma gondii* in Wild Mammals of Southern Italy. Pathogens.

[23] Rostami A., Karanis P., Fallahi S. (2018). Advances in Serological, Imaging Techniques and Molecular Diagnosis of *Toxoplasma gondii* Infection. Infection.

[24] Holec-Gasior L. (2013). *Toxoplasma gondii* Recombinant Antigens as Tools for Serodiagnosis of Human Toxoplasmosis: Current Status of Studies. Clin. Vaccine Immunol..

[25] Gamble H.R., Andrews C.D., Dubey J.P., Webert D.W., Parmley S.F. (2000). Use of Recombinant Antigens for Detection of *Toxoplasma gondii* Infection in Swine. J. Parasitol..

[26] Jiang T., Gong D., Ma L.A., Nie H., Zhou Y., Yao B., Zhao J. (2008). Evaluation of a Recombinant MIC3-Based Latex Agglutination Test for the Rapid Serodiagnosis of *Toxoplasma gondii* Infection in Swine. Vet. Parasitol..

[27] Ismael A.B., Sekkai D., Collin C., Bout D., Mévélec M.N. (2003). The MIC3 Gene of *Toxoplasma gondii* Is a Novel Potent Vaccine Candidate against Toxoplasmosis. Infect. Immun..

[28] Yang Y., Huang Y., Zhao X., Lin M., Chen L., Zhao M., Chen X., Yang Y., Ma G., Yao C. (2022). Development of an Immunochromatographic Test Based on Rhoptry Protein 14 for Serological Detection of *Toxoplasma gondii* Infection in Swine. Animals.

[29] Ferra B., Holec-Gąsior L., Kur J. (2015). Serodiagnosis of *Toxoplasma gondii* Infection in Farm Animals (Horses, Swine, and Sheep) by Enzyme-Linked Immunosorbent Assay Using Chimeric Antigens. Parasitol. Int..

[30] Fan J., Sun H., Fang J., Gao Y., Ding H., Zheng B., Kong Q., Zhuo X., Lu S. (2024). Application of Gold Immunochromatographic Assay Strip Combined with Digital Evaluation for Early Detection of *Toxoplasma gondii* Infection in Multiple Species. Parasites Vectors.

[31] Song K.J., Yang Z., Chong C.K., Kim J.S., Lee K.C., Kim T.S., Nam H.W. (2013). A Rapid Diagnostic Test for Toxoplasmosis Using Recombinant Antigenic N-Terminal Half of SAG1 Linked with Intrinsically Unstructured Domain of GRA2 Protein. Korean J. Parasitol..

[32] Hill D., Dubey J.P. (2002). *Toxoplasma gondii*: Transmission, Diagnosis and Prevention. Clin. Microbiol. Infect..

[33] Weiss L.M., Dubey J.P. (2009). Toxoplasmosis: A History of Clinical Observations. Int. J. Parasitol..

[34] Lourenço E., Bernardes E., Silva N., Mineo J., Panunto-Castelo A., Roque-Barreira M. (2006). Immunization with MIC1 and MIC4 Induces Protective Immunity against *Toxoplasma gondii*. Microbes Infect..

