# Supplementary figures and images for: Development of an Immunochromatographic Test with Recombinant MIC2-MIC3 Fusion Protein for Serological Detection of Toxoplasma gondii
Source: Vet Sci. 2025 May 22;12(6):509. doi: 10.3390/vetsci12060509 (PMC12197616; doi:10.3390/vetsci12060509)

Original WB

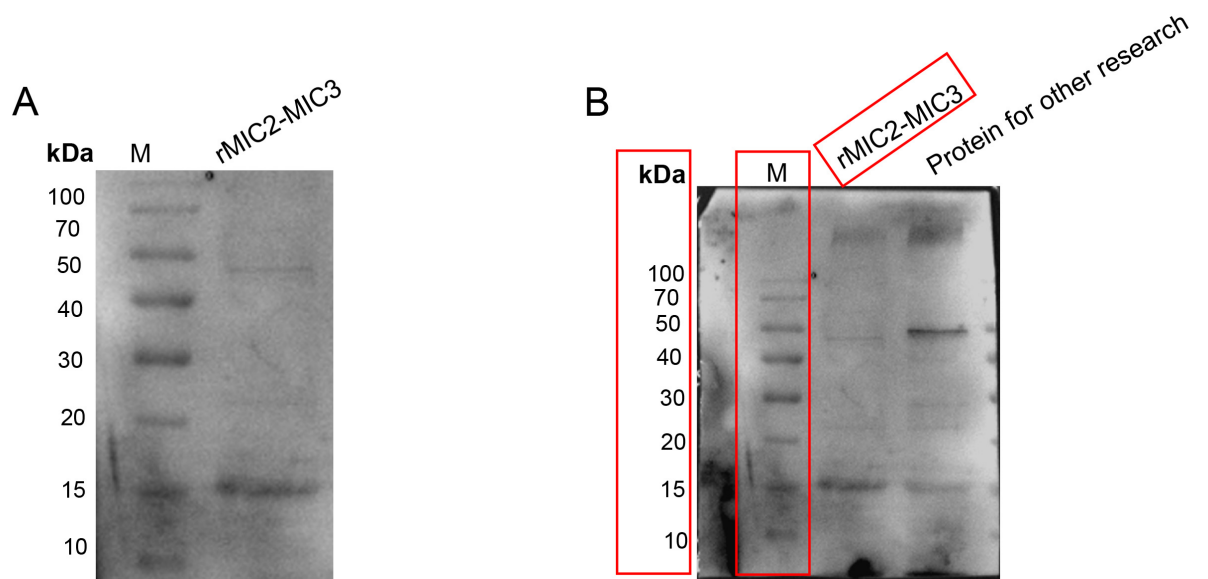

Figure S1 western-blot analysis for recombinant protein rMic2-Mic3

Supplement: Supplementary file 1 [file vetsci-12-00509-s001.zip › vetsci-3603747-supplementary.pdf]
